# Skewed Lung CCR4 to CCR6 CD4^+^ T Cell Ratio in Idiopathic Pulmonary Fibrosis Is Associated with Pulmonary Function

**DOI:** 10.3389/fimmu.2016.00516

**Published:** 2016-11-23

**Authors:** Ayodeji Adegunsoye, Cara L. Hrusch, Catherine A. Bonham, Mohammad R. Jaffery, Kelly M. Blaine, Meghan Sullivan, Matthew M. Churpek, Mary E. Strek, Imre Noth, Anne I. Sperling

**Affiliations:** ^1^Section of Pulmonary and Critical Care, Department of Medicine, University of Chicago, Chicago, IL, USA; ^2^Committee on Immunology, University of Chicago, Chicago, IL, USA

**Keywords:** CD4, idiopathic pulmonary fibrosis, chemokine receptors, T cells, lung function

## Abstract

**Rationale:**

Idiopathic pulmonary fibrosis (IPF) is a progressive, fatal lung disease. While it has been suggested that T cells may contribute to IPF pathogenesis, these studies have focused primarily on T cells outside of the pulmonary interstitium. Thus, the role of T cells in the diseased lung tissue remains unclear.

**Objective:**

To identify whether specific CD4^+^ T cell subsets are differentially represented in lung tissue from patients with IPF.

**Methods:**

CD4^+^ T cell subsets were measured in lung tissue obtained from patients with IPF at the time of lung transplantation, and from age- and gender-matched organ donors with no known lung disease. Subsets were identified by their surface expression of CCR4, CCR6, and CXCR3 chemokine receptors. CD4^+^ T cell subsets were correlated with measurements of lung function obtained prior to transplantation.

**Results:**

Compared to controls, IPF patients had a higher proportion of lung CD4^+^ T cells, a higher proportion of CCR4^+^ CD4^+^ T cells, and a lower proportion of CCR6^+^ CD4^+^ T cells. The increase in CCR4^+^ CD4^+^ T cells in IPF lung tissue was not due to increased Tregs. Intriguingly, the increase in the ratio of CCR4^+^ cells to CCR6^+^ cells correlated significantly with better lung function.

**Conclusion:**

Our findings suggest a new paradigm that not all T cell infiltrates in IPF lungs are detrimental, but instead, specialized subsets may actually be protective. Thus, augmentation of the chemokines that recruit protective T cells, while blocking chemokines that recruit detrimental T cells, may constitute a novel approach to IPF therapy.

**Scientific Knowledge on the Subject:**

Idiopathic pulmonary fibrosis (IPF) is a progressive pulmonary disease with a poor prognosis. The etiology of the disease is unknown; however, recent studies have found that immunosuppression and decreased expression of T cell co-stimulatory pathways in IPF are linked to reduced survival. Insight into mechanisms involving pulmonary T cell populations may improve understanding of IPF pathogenesis and lead to development of targeted therapies.

**What This Study Adds to the Field:**

We show that CD4^+^ T cells are increased and that a specific ratio of phenotypically distinct subsets of CD4^+^ T cells is found in IPF lung parenchyma and lung lymph nodes. Interestingly, increased pulmonary CCR4 to CCR6 ratios correlated with higher percentage forced vital capacity, implying a positive effect of this skewed ratio in the preservation of lung function. Our study illuminates a complex role for adaptive immunity in IPF pathogenesis.

## Introduction

Idiopathic pulmonary fibrosis (IPF) is a chronic, progressive, and fatal interstitial lung disease of unknown cause (1). It is characterized by excess collagen deposition and parenchymal fibrosis in the lungs (2), and the ensuing impairment of respiratory function and gas exchange is associated with significant morbidity and mortality. Fibroblast activation, matrix deposition, and lung remodeling all contribute to IPF pathogenesis (3, 4). Circulating fibrocytes derived from the bone marrow contribute to pulmonary fibrosis by producing collagen, secreting cytokines that promote collagen deposition, and differentiating into fibroblasts (5–7). Chronic activation of fibroblasts alters their phenotype leading to the formation of myofibroblasts, which are associated with overproduction of collagen matrix. Cytokines like TGF-β and platelet-derived growth factor (PDGF) produced by these alveolar epithelial cells further promote fibroblast proliferation (8, 9). Recently, the anti-fibrotic medications nintedanib and pirfenidone were approved for the treatment of IPF (10–12) through the inhibition of tyrosine kinases and TGF-β, respectively. However, while these therapies appear to slow disease progression, they do not halt the decline in lung function.

Whether the adaptive immune system contributes to IPF etiology and pathology remains controversial (13–16). Patients with IPF often have an increased pulmonary bacteria burden, and some acute exacerbations of IPF have been linked to viruses (17). This increased microbial burden may be linked to the augmentation of activated circulating leukocyte populations in IPF patients (18). Further, a recent trial showed that suppression of immune cell function in IPF patients could be harmful (19). Models for genetic prognostic prediction in IPF indicate an involvement of T cell pathways, and in murine studies, T cells influence the development of pulmonary fibrosis (20, 21). Although the exact effects of T cell cytokines on myofibroblasts and ECM deposition remain unclear, Th1, Th22, and γδ-T cells are classically thought to attenuate fibrosis (22, 23) while those linked to Th2 and Th17 have been linked to fibrogenesis (20, 24–26). Furthermore, IPF patients with increased percentages of circulating CXCR3^+^ CD4^+^ and CXCR3^+^ CD8^+^ T cells have reduced progression-free survival, suggesting a role for T-lymphocyte trafficking in IPF progression (27). Depending on the stage of pulmonary fibrosis and their interactions with other T cell subtypes, regulatory T cells (Tregs) may exert anti- or pro-fibrotic roles (21, 28). Together, these studies highlight the complex pathways through which T cells may modulate fibrogenesis; however, most studies have been performed on peripheral blood, BAL, or in mouse models, and the contribution of T cells to the pathology and fibrosis in the lung parenchyma remains unknown (29).

Herein, we demonstrate an increase of CCR4^+^ CD4^+^ T cells and a reduction in CCR6^+^ CD4^+^ T cells in the lung tissue and lung lymph nodes (LLN) of patients with IPF at the time of lung transplantation compared to non-transplantable lung and lymph nodes from control donors. Strikingly, the ratio of pulmonary CCR4^+^ to CCR6^+^ CD4^+^ T cells highly correlates with forced vital capacity (FVC) measured in the IPF patients, linking these CD4^+^ subsets to lung function. Our novel findings imply that the recruitment and expansion of specific chemokine receptor positive CD4^+^ T cells can directly preserve lung function, thereby impacting disease progression.

## Materials and Methods

### Subjects and Tissue Samples

Tissue samples were obtained from two sources. Study participants with IPF were recruited from the University of Chicago Lung Transplantation program after informed consent had been obtained under institutional review board protocol 14514A. A multidisciplinary diagnosis of IPF was made in accordance with the American Thoracic Society/European Respiratory Society 2011 guidelines (1). Participants were screened at Transplant Clinic visits during which pulmonary function tests and clinical and laboratory data were obtained, and these patients were subsequently deemed eligible for lung transplantation. Blood was obtained from recruited subjects with IPF immediately prior to lung transplantation (Figure S1 in Supplementary Material). Recruited subjects with IPF also donated their explanted lungs and LLN at the time of lung transplantation. Non-transplantable control lungs and LLN were obtained from the Gift of Hope/Regional Organ and Tissue Donor Network (GOH) organ and tissue donor network, an organ procurement organization that provides services regionally to 12 million people within the national donation system. Subjects from whom control specimens (GOH) were obtained had no known history of lung disease or immunologic disorder. While the exact reason for rejecting each donor lung was unknown, donor lungs utilized in this study were deemed non-transplantable by the lung transplant team for various reasons according to standard pre-transplantation criteria, such as lung size mismatch, older donor age, and duration of smoking history.

### Tissue Processing and Cell Preparation

Peripheral blood mononuclear cells (PBMCs) were isolated from heparinized blood by utilizing density gradient centrifugation with Histopaque-1077 (Sigma-Aldrich). To obtain lung leukocytes from IPF or control lungs, lung tissue was minced using dissection scissors to an approximate size of 2 mm^3^. Minced tissue was digested in RPMI media containing 10% FBS, 240 U/mL Collagenase IV (Sigma-Aldrich), and 4 μg/mL DNase I (Worthington Biochemical Corporation, Lakewood, NJ, USA) at 37°C for 90 min. Digested tissue was filtered through nylon mesh, and mononuclear cells were enriched by gradient centrifugation using Histopaque-1077 (Sigma-Aldrich). LLN cells were extracted from multiple hilar lymph nodes by mechanical dissociation. Isolated cells from the blood, lung, and LLN from IPF and control tissues were cryopreserved until analysis.

### Flow Cytometry

Cells were surface-stained with anti-CD3-FITC, anti-CD4-AlexaFluor700, anti-CD8-PE/Dazzle594, anti-CD127-PE-Cy7, anti-CCR6-BrilliantViolet421, anti-CCR4-PE, and anti-CXCR3-PerCP/Cy5.5 (Biolegend). Cells were fixed and permeabilized to stain for the intracellular transcription factor FoxP3 using anti-FoxP3-AlexaFluor647 according to manufacturer’s instructions (eBioscience). Cell fluorescence was measured using a LSR II Fortessa flow cytometer (BD Biosciences) and analysis was performed using FlowJo software (Tree Star).

### Statistical Analysis

The distributions of all measured variables were analyzed using non-parametric testing, Student’s *t*-test, or Pearson’s correlation where appropriate. Group matching of controls to cases was performed based on age (±5 years), gender, and race. Demographic continuous variables were analyzed using paired *t*-test analysis, while comparisons of median cell proportions were performed using Mann–Whitney *U* analysis. Lung function tests and cell proportions were correlated using the Pearson correlation test. Statistical significance was determined using a two-sided *p-*Value of less than 0.05. All statistical analyses were performed using GraphPad Prism (La Jolla, CA, USA).

## Results

We evaluated a cohort comprised of 9 lungs from subjects with IPF and 13 control lungs from donors with no known lung disease. Baseline clinical characteristics of the study cohorts were not significantly different between groups (Table 1).

**Table 1:** **Characteristics of study subjects**.

Characteristic	IPF (*n* = 9)	GOH (*n* = 13)	*p*-Value
Age, mean (SD)	58.7 (7.6)	58.0 (4.1)	0.78
Male, *n* (%)	6 (67)	4 (31)	0.19
Race/ethnicity			
White, *n* (%)	6 (67)	8 (62)	1.00
Non-white, *n* (%)	3 (33)	5 (37)	1.00
Ever smoker, *n* (%)	6 (67)	7 (54)	0.67
Pack years, mean (SD)	28.2 (25.6)	16.9 (12.9)	0.33
Immunosuppressive therapy, *n* (%)	2 (22)	2 (15)	1.00
Diabetes, *n* (%)	1 (11)	4 (31)	0.36

*p values for age and pack years were determined by utilizing the t-test. Other p values were computed using Pearson’s chi-squared or two-sided Fisher’s exact test where applicable*.

### Lung Tissue CD4^+^ T Cells Are Increased in IPF Patients

**Figure 1:**
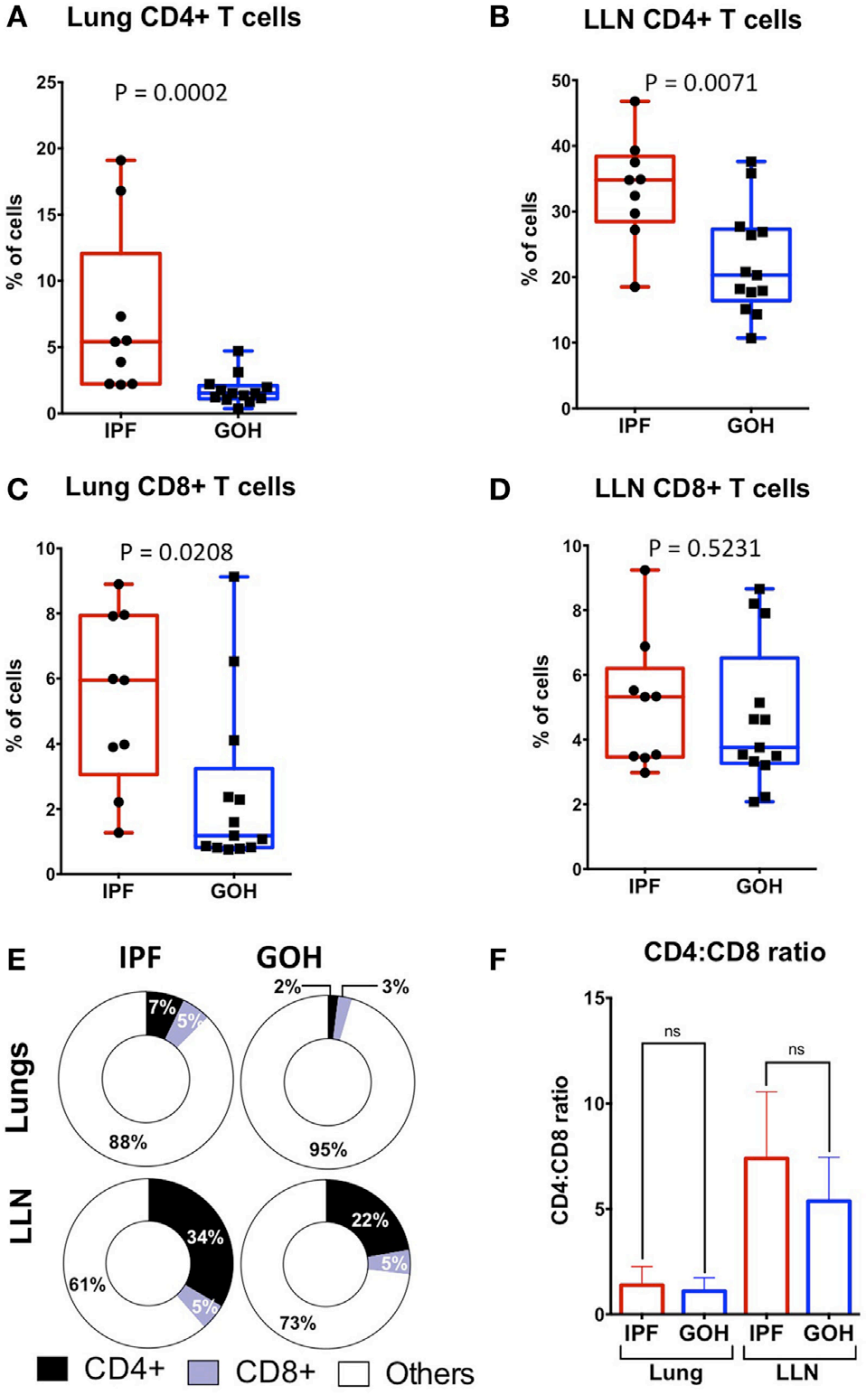
**CD4^+^ T cells constitute a greater percentage of tissue cells in IPF lungs and lung lymph nodes (LLN) than in non-IPF controls (GOH)**. CD4^+^ T cells are increased in IPF lungs **(A)**; and LLN **(B,E)** compared to non-IPF controls. However, CD8^+^ T cell percentages are increased in IPF lungs **(C)**, but similar in the LLN **(D,E)**; while CD4:CD8 ratios are similar **(F)**. Significance determined by Mann–Whitney test. Mean values depicted **(E)**.

Substantial variations in the proportions of circulating CD4^+^ T cells in IPF patients may not accurately reflect their proportions in lung tissue (27, 30). To elucidate the pattern of CD4^+^ T cell distribution in IPF lungs and LLN, we analyzed the absolute percentages of CD4^+^ T cells and CD8^+^ T cells in tissues from subjects with IPF and from controls. The median percentage of CD4^+^ T cells in lung tissue of subjects with IPF (5.40%; range, 2.17–19.10%) was significantly higher than in controls (1.53%; range, 0.37–4.70%; *p* = 0.0002) (Figure 1A). Similarly, the median percentage of CD4^+^ T cells in LLN of subjects with IPF (34.80%; range, 18.50–46.80%) was significantly higher than in controls (20.30%; range, 10.70–37.60%; *p* = 0.007) (Figure 1B). While no significant difference was observed in the percentages of CD8^+^ T cells in LLNs of subjects with IPF compared to controls (*p* = 0.523), the median percentage of CD8^+^ T cells in lung tissue of subjects with IPF (5.95%; range, 1.27–8.90%) was higher than in controls (1.18%; range, 0.75–9.13%; *p* = 0.021) (Figures 1C,D). The dramatic increase found in the proportion of CD4^+^ T cells was likely not due to a decrease in another cell type since the CD4^+^ T cells were only a small fraction of total lung cells (Figure 1E). No significant difference was observed in the CD4:CD8 T cell ratio in the lungs (*p* = 0.467) or LLNs (*p* = 0.131) (Figure 1F). While we were unable to obtain blood from our controls, the IPF patient’s blood had significantly greater CD4^+^ T cells (35.95%; range, 24.70–44.20%) than CD8^+^ T cells (19.99%; range, 10.50–33.10%; *p* = 0.002), and a CD4:CD8 ratio of 2.15 (range, 0.81–3.58). Thus, the percentages of CD4^+^ T cells are dramatically increased in both the lung and LLNs of IPF patients, suggesting that they either undergo expansion specifically in IPF tissues, or are highly recruited from the circulating pool of CD4^+^ T cells.

### Chemokine Receptor Expression on CD4^+^ T Cells from the Lungs Differs from LLNs and Peripheral Blood

Human CD4^+^ T cells express chemokine receptors that drive their migration from the blood to the tissues based on gradients of specific chemokines (31, 32). In the blood, specific Th subsets can be identified by the expression of chemokine receptors on CD4^+^ T cells (33): CCR4^+^ (CCR6^−^) CD4^+^ T cells are associated with Th2 cells, CXCR3^+^ (CCR4^−^ CCR6^−^) CD4^+^ T cells are associated with Th1 cells, CCR6^+^ (CXCR3^−^) CD4^+^ T cells are associated with Th17 cells, and CCR6^+^ CXCR3^+^ CD4^+^ T cells produce both Th1 and Th17 cytokines (hereafter referred to as CCR4^+^, CXCR3^+^, CCR6^+^, or CCR6^+^CXCR3^+^CD4^+^ T cells, respectively).

**Figure 2:**
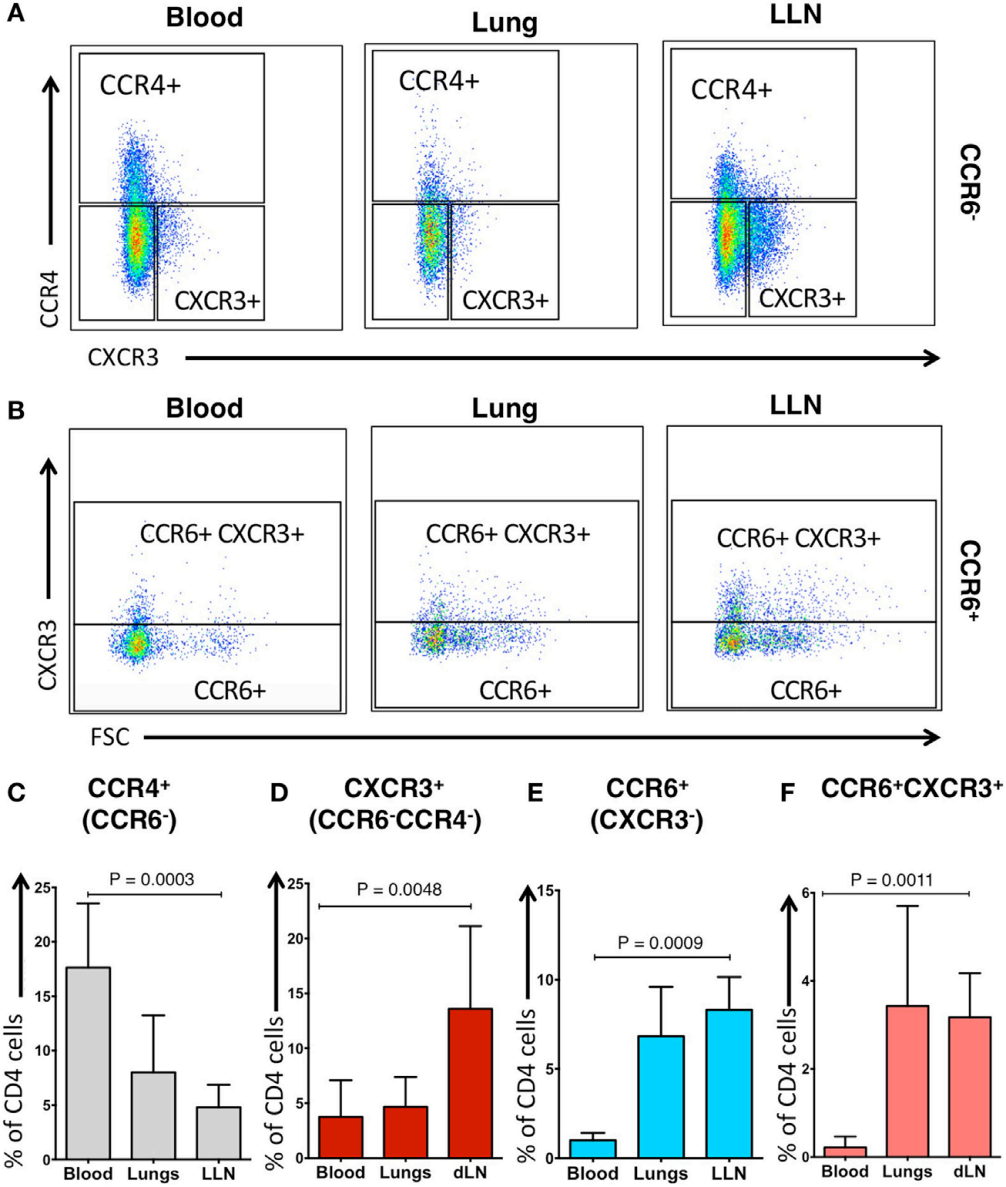
**Phenotypic distribution of CD4^+^ CCR6^−^ T cells and CD4^+^ CCR6^+^ T cells differ across tissue locations**. CCR6^−^ blood cells have a predominant pattern of CCR4 expression **(A,C)** while CCR6^−^ lung lymph nodes (LLN) cells have predominant pattern of CXCR3 expression **(A,D)**. CCR6^+^ blood cells have reduced CXCR3 expression **(B,E)** while CCR6^+^ LLN cells have increased CXCR3 expression compared to blood **(B,F)**. Peripheral blood of IPF subjects demonstrates higher proportion of CCR4^+^CCR6^−^CD4 T cells **(C)**, but lower proportion of CCR6^+^CXCR3^−^ cells when compared to the lungs **(E)**. Significance determined by the Friedman non-parametric paired ANOVA with *post hoc* Dunn test.

Using an established gating strategy for blood CD4^+^ T cells (Figure S2 in Supplementary Material), CCR6, CCR4, and CXCR3 expression on CD4^+^ T cells was determined in these compartments (Figure 2). CCR4^+^ CD4^+^ T cells within the blood of our IPF cohort (mean, 17.63 ± 5.91%) was significantly higher than in the lung tissue (mean, 8.01 ± 5.23%; *p* = 0.0003) (Figures 2A,C). The proportion of CCR6^+^ CD4^+^ T cells within the blood of our IPF cohort (mean, 1.00 ± 0.42%) was significantly decreased when compared to the proportion of lung and LLN CCR6^+^ CD4^+^ T cells (mean, 6.83 ± 2.78%; and mean, 8.31 ± 1.84%; *p* = 0.0009, respectively) (Figures 2B,E). Unlike cells from the blood or lungs, LLN CD4^+^ T cells demonstrated a significantly higher expression of CXCR3 (Figures 2A,D). The proportion of CCR6^+^ CXCR3^+^ CD4^+^ T cells within the blood of our IPF cohort (mean, 0.22 ± 0.25%) was significantly decreased when compared to the proportion of lung and LLN CCR6^+^ CXCR3^+^ CD4^+^ T cells (mean, 3.43 ± 2.27%; and mean, 3.18 ± 1.00%; *p* = 0.0011, respectively) (Figures 2B,F). While we did not have access to blood from the control donors, between the lungs, and LLN from the control donors, there was only a small difference in the CXCR3^+^ CD4^+^ T cells (Figure S3 in Supplementary Material). Thus, substantial differences in chemokine receptor expression on CD4^+^ T cells were found, based on their tissue location.

### CCR4^+^ CD4^+^ T Cells Are Increased in the Lungs of IPF Patients Compared to Control Lungs

**Figure 3:**
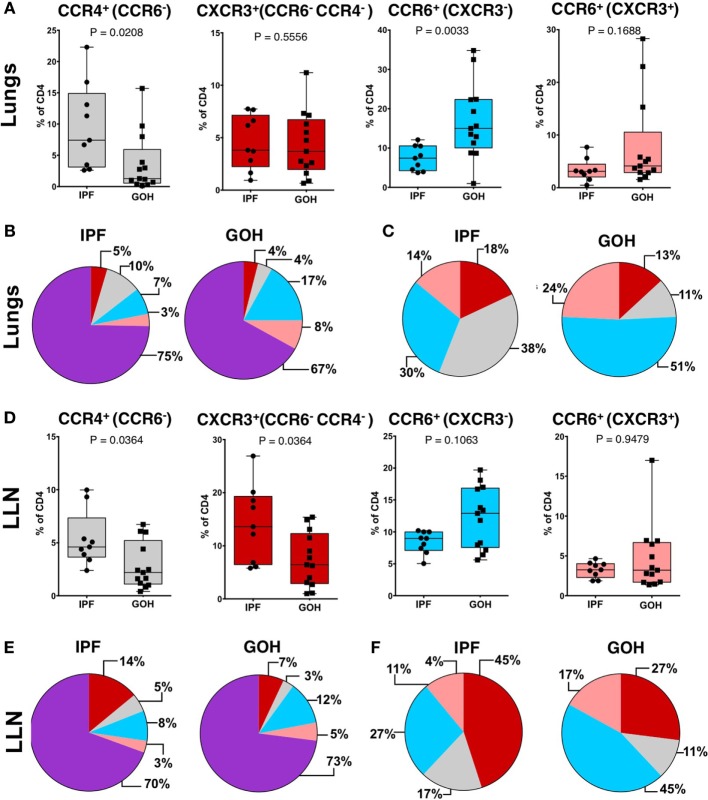
**CCR4^+^CCR6^−^ cells are the predominant subset in IPF lungs while CXCR3^−^CCR6^+^ cells are the predominant subset in non-IPF lungs (GOH)**. After separation of CD3^+^CD4^+^ T cells into CCR6^−^ or CCR6^+^ cells, these subsets were then divided into CXCR3^+^CCR4^−^CCR6^−^ cells, CXCR3^−^CCR4^+^CCR6^−^ cells, CXCR3^−^CCR6^+^ cells, or CXCR3^+^CCR6^+^ cells as shown **(A,B)**. The majority of cells were unpolarized (*purple*) in both groups **(C)**. Among the polarized subsets, CCR6^−^CCR4^+^ cells (*gray*) were the majority in IPF while CCR6^+^CXCR3^−^ cells (*blue*) were the majority in non-IPF **(D)**. In IPF lungs, CCR6^−^CCR4^+^ cells were higher, CXCR3^+^CCR4^−^CCR6^−^ cells were not different, and both CXCR3^−^CCR6^+^ and CXCR3^+^CCR6^+^ cells were lower compared to controls **(E)**. Significance determined by Mann–Whitney test. Mean values depicted **(B,C,E,F)**.

To understand the role of CD4^+^ T cells within the fibrotic lungs, we evaluated the proportions of CD4^+^ T cell subsets as defined by their chemokine receptors. A striking increase in the percentage of CCR4^+^ CD4^+^ T cells was found in the IPF lungs (7.42%; range, 2.60–22.30%), in sharp contrast to control (GOH) lungs (1.28%; range, 0.15–15.70%; *p* = 0.021) (Figure 3A). Conversely, CCR6^+^ T cells in the lungs of IPF subjects were decreased (7.43%; range, 3.74–12.10%) in comparison to controls (15.00%; range, 0.98–34.80%; *p* = 0.003). There were no significant differences in the proportions of CXCR3^+^ CD4^+^ T cells (*p* = 0.551) or CCR6^+^ CXCR3^+^ CD4^+^ T cells (*p* = 0.169) between the two groups. As the majority of CD4^+^ T cells in the lungs do not express any of the tested chemokine receptors, the increase in the CCR4^+^ CD4^+^ T cells was not due to the decrease in the CCR6^+^ CD4^+^ T cells, and vice versa (Figure 3B). When we focused only on the chemokine receptor-expressing CD4^+^ T cells, CCR4^+^ CD4^+^ T cells were found to constitute the greatest percentage in IPF lungs, while CCR6^+^ CD4^+^ T cells constituted the greatest percentage in control lungs (Figure 3C). As the percentage of CCR7^+^ CD4^+^ cells in the lungs did not differ between IPF and control lungs, not all chemokine receptors are affected in IPF lungs (Figure S4 in Supplementary Material).

**Figure 4:**
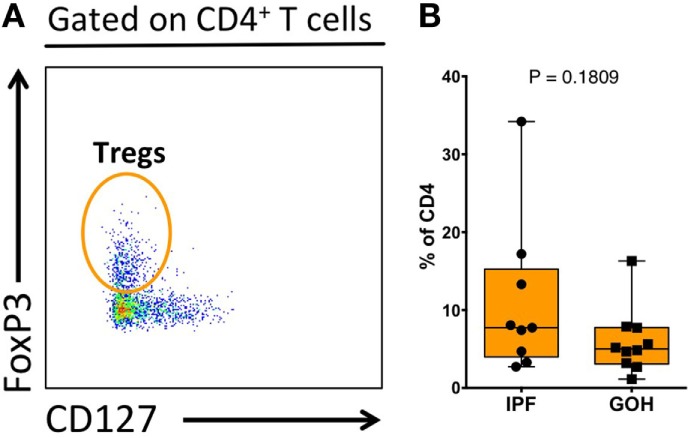
**Proportion of regulatory T cells (Tregs) within total lung CD4^+^ T cells**. Tregs were identified as CD3^+^CD4^+^FoxP3^+^CD127^−^ cells **(A)**. The proportion of Tregs does not differ between IPF and non-IPF controls (GOH) **(B)**. Significance determined by Mann–Whitney test.

Similar to lung tissue, CCR4^+^ CD4^+^ T cells in the LLNs were increased (4.61%; range, 2.39–9.98%) compared to control LLNs (2.20%; range, 0.41–6.73%; *p* = 0.036) (Figure 3D). Although the CCR6^+^ CD4^+^ T cells were not significantly different between the two groups, there was a trend toward decreased percentages in the IPF LLNs. However, unlike CD4^+^ T cells within the lungs, the proportion of CXCR3^+^ CD4^+^ T cells was higher in IPF LLNs (13.60%; range, 5.80–26.90%) compared to controls (6.43%; range, 1.00–15.40%; *p* = 0.036). No differences in the percentages of CCR7^+^ CD4^+^ T cells were found in the lungs or LLN (Figure S4 in Supplementary Material). When focusing only on T cells positive for any of the three chemokine receptors, CXCR3^+^ CD4^+^ T cells constituted the greatest percentage in IPF LLNs, while CCR6^+^ CD4^+^ T cells constituted the greatest percentage in controls (Figures 3E,F). CCR4 is a marker of both conventional and regulatory T cells (Tregs). However, no differences were found in Tregs proportions (*p* = 0.181) (Figure 4) suggesting that the increase in CCR4^+^ CD4^+^ T cells in the lungs is not primarily due to increased Tregs.

### Increased Ratio of Lung CCR4^+^ to CCR6^+^ CD4^+^ T cells Correlates with Better Forced Vital Capacity in IPF

**Figure 5:**
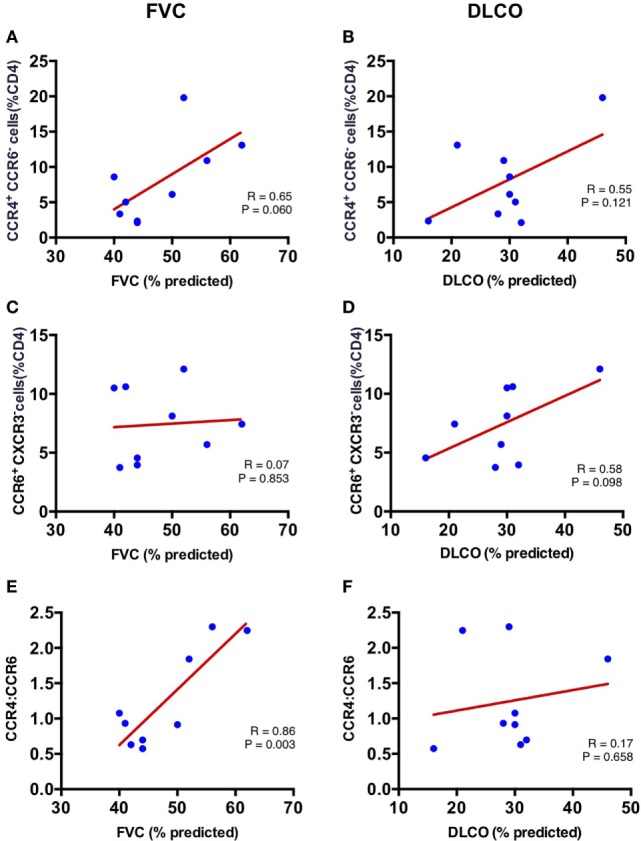
**Correlation of FVC and DLCO with CCR4^+^ CCR6^−^ percent of CD4^+^ T cells and CCR6^+^ (CXCR3^−^) percent of CD4^+^ T cells in the lungs at time of lung explant**. Lung CCR4^+^CCR6^−^ percent of CD4^+^ T cells correlates with percentage forced vital capacity (FVC%) (*r* = 0.646; *p* = 0.061) **(A)**; but not percentage diffusion capacity for carbon monoxide (DLCO%) (*r* = 0.555; *p* = 0.121) **(B)**. Lung CCR6^+^ (CXCR3^−^) percent of CD4^+^ T cells does not correlate with FVC% (*r* = 0.073; *p* = 0.853) **(C)**; or DLCO% (*r* = 0.585; *p* = 0.031) **(D)**. The ratio of lung CCR4^+^/CXCR3^−^ strongly correlates with percentage forced vital capacity (FVC%) (*r* = 0.863; *p* = 0.003) **(E)**; but not with DLCO% (*r* = 0.172; *p* = 0.658) **(F)**.

To determine whether the skewed populations of CD4^+^ T cells in the IPF lungs are related to disease pathogenesis, we evaluated whether the percentages of lung CCR4^+^ and CCR6^+^ CD4^+^ T cells correlated with pulmonary function in our IPF cohort. Pulmonary function indices utilized were the most recent pretransplant percent predicted FVC, and percent predicted diffusion capacity for carbon monoxide (DLCO). The FVC demonstrated a positive correlation with lung CCR4^+^ CD4^+^ T cells (*r* = 0.65) although this did not reach statistical significance (*p* = 0.06) (Figure 5A), while the DLCO demonstrated no significant correlation with lung CCR4^+^ CD4^+^ T cells (*r* = 0.56; *p* = 0.12) (Figure 5B). In contrast, the proportion of CCR6^+^ CD4^+^ T cells in lung tissue of IPF patients trended with DLCO (*r* = 0.58, *p* = 0.10), but not with FVC (*r* = 0.07; *p* = 0.85) (Figures 5C,D). No other CD4^+^ subset demonstrated significant correlation with FVC or DLCO. In contrast with the lungs, the CD4^+^ T cell subsets from LLN and blood did not correlate with lung function measures, suggesting that the lung CD4^+^ T cells are primarily involved in IPF pathogenesis (Table S1 in Supplementary Material).

As both lung CCR4^+^ and CCR6^+^ CD4^+^ T cells differed in proportion in the IPF patients (Figure 3A), we hypothesized that the ratio of these two cell types in each IPF patient may provide a more stable predictor of the extent of disease. Strikingly, the CCR4 to CCR6 ratio was significantly associated with FVC in our cohort (*r* = 0.863; *p* = 0.003) (Figure 5E), although no association was found with DLCO (*r* = 0.172; *p* = 0.658) (Figure 5F). These results imply that the increase in CCR4^+^ CD4^+^ T cells, and decrease in CCR6^+^ CD4^+^ T cells within the lungs of IPF patients may directly influence preservation of lung function.

## Discussion

Our study suggests a complex role for adaptive immunity in IPF pathogenesis. We report that CD4^+^ T cells are increased in IPF lung parenchyma and LLN, and that a specific ratio of phenotypically distinct subsets of CD4^+^ T cells is found in IPF patients. We investigated three prominent chemokine receptors expressed on CD4^+^ T cells and found that IPF patients have a skewed composition with an increased proportion of lung CCR4^+^ CD4^+^ T cells and a decreased proportion of CCR6^+^ CD4^+^ T cells. Moreover, we present the first evidence that an increased ratio of CCR4^+^ to CCR6^+^ CD4^+^ T cells in lung tissue of IPF patients is significantly associated with higher percent predicted FVC, implying a positive effect of this skewed ratio in the preservation of lung function. Prominent clinical trials such as PANTHER have demonstrated worse outcomes in patients receiving a combination of immunosuppressive agents (34). The poor results of generalized immunosuppression in IPF can be newly examined in light of our findings that some subsets of lung T cells may be protective. Our study suggests that a better approach to treatment could be targeting the specific T cell subsets that are harmful or beneficial in IPF, for example, augmenting CCR4^+^ CD4^+^ T cell recruitment to the lungs while blocking CCR6^+^ CD4^+^ T cells.

The involvement of adaptive immunity in IPF is controversial. Our study showed a dramatic increase in CD4^+^ T cells in both the lung and LLN of patients, which supports the concept that the adaptive immune system is involved in IPF (18, 27). Unlike the lungs, circulating CD4^+^ T cells are present in similar numbers in IPF patients and controls, or are even decreased in IPF patients (27, 30). This finding, in conjunction with the upregulation of specific chemokine receptors in IPF patients, suggests that peripheral cells may be actively trafficking to the site of disease. Interestingly, while CD4^+^ T cells were increased in both IPF lung and LLN, CD8^+^ cells were found in significantly greater numbers in lung tissue, but not LLN. This raises the question of altered CD8^+^ cell trafficking; perhaps between various naive, memory, and effector CD8^+^ T cells. Others have demonstrated antigen-independent localization of memory CD8 T cells to the lung following viral infection (35, 36), with loss of CD62L expression preventing return of activated effector CD8^+^ T cells to the LLN (37). Furthermore, both exposure to high levels of inflammatory cytokines (38) and presence of chronic infections such as HIV, EBV, and CMV (39) have been shown to impair effector CD8^+^ T cell trafficking back to lymph nodes. In our study, IPF patients presenting for transplant had experienced previous deteriorations in pulmonary function, which may provide the stimulus for altered CD8^+^ mucosal memory at the time of their transplant.

CCR4 expression allows T cells to migrate in response to multiple chemokines, including CCL2, a chemokine that is released from epithelium adjacent to fibrotic areas in IPF lungs. CCR4 is a chemokine receptor expressed on both Th2 and Tregs. However, we found no difference in the proportion of Tregs in the lung parenchyma in IPF patients, suggesting that the increased CCR4 CD4 T cells may be Th2 cells and not Tregs. While peripheral Tregs have been reported to increase during IPF disease progression (16, 27, 40), our study is consistent with others that have reported no change in Tregs within the lung interstitium of IPF patients (41, 42). Thus, we hypothesize that the increased CCR4^+^ CD4^+^ T cells may be Th2 cells, not Tregs. Th2 responses have been widely implicated in both tissue repair and development of fibrosis (43, 44), leading most to infer that pro-fibrotic Th2 responses would be detrimental to IPF. As interferon gamma (IFNγ) is known to inhibit Th2 cytokine production, the failure of a large clinical trial treating IPF patients with IFNγ provides doubt about whether Th2 responses are inherently disadvantageous to IPF (45). However, we notably find that increased lung CCR4^+^ CD4^+^ T cells positively correlate with lung function in IPF. This suggests a model in which pro-fibrotic Th2 responses play a paradoxically beneficial role in IPF, perhaps by conferring greater resiliency to intermittent lung injury. Consistent with our findings, a protective role for Th2 immunity in tissue repair and homeostasis has been recognized in other disease processes (46, 47).

CCR6^+^ CD4^+^ T cells were reduced in IPF lungs in comparison to controls, and the ratio of CCR4^+^ to CCR6^+^ CD4^+^ T cells correlated with improved lung function, suggesting that CCR6^+^ CD4^+^ T cells in IPF lungs negatively impact pulmonary function. While it has not been confirmed in lung tissue, in the blood, CCR6 is associated with Th17 CD4^+^ T cells (33). Th17 cells influence disease progression in murine models of pulmonary fibrosis (20, 48), and in IPF patients, increased IL-17 in the bronchoalveolar lavage (BAL) (20) and decreased Th17 cells in blood samples (40) have been found. Our finding of an elevated CCR4^+^/CCR6^+^ ratio in patients with higher lung function supports a model in which Th17 responses produced by lung resident CCR6^+^ CD4^+^ cells are potentially harmful. As IL-17 is associated with human airway neutrophil recruitment (49), and BAL neutrophilia predicts early mortality in IPF (50), our findings of decreased CCR6^+^ CD4^+^ T cells in the lungs may protect from detrimental neutrophilia. Thus, further study of neutrophilic, pro-inflammatory, and pro-fibrotic Th17 responses, as well as confirmation of the Th17 identity of the CCR6^+^ CD4^+^ cells within the IPF lung, is warranted to understand the impact of Th17 polarization upon IPF disease progression.

The chemokine receptor CXCR3 is associated with Th1 cells that produce IFN-γ, which has been linked to attenuation of fibrogenesis in mouse models (51), but not human clinical trials (45). It has previously been demonstrated that IPF patients have reduced CXCR3 expression on BAL CD4^+^ T cells compared to controls (52). In contrast, in our cohort, no decrease in CXCR3^+^ CD4^+^ T cells was observed in the lung parenchyma, and IPF patients actually exhibited increased CXCR3^+^ CD4^+^ T cells in the LLN compared to controls. As IPF is an interstitial disease, the BAL studies may not be representative of the underlying process within the lung parenchyma, and therefore, the subsets of leukocytes obtained from BAL may be very different compared to tissue-resident subsets (53, 54).

Our study had a number of limitations. Given the limited number of explanted IPF lungs available to us, our findings may not be powered to identify all potential effects. Studies with more patients are needed to explore the effects of chemokine receptor signaling pathways in IPF. Further, by utilizing explanted lungs from patients with end-stage lung disease, our data may not be representative of IPF patients earlier in their disease course, therefore, these results may not be applicable to all patients with IPF. Another limitation of our study is the lack of live/dead stain for flow cytometry. However, we carefully selected our live cells by FSC/SSC gating, and none of the analyses performed on our samples involved rare cell populations. Last, while chemokine receptor-expressing CD4^+^ T cell subsets have been well described in the peripheral blood, it is unknown if tissue-derived CD4^+^ T cell subsets can also be functionally characterized using chemokine receptor expression.

For the first time, these results link lung parenchymal CD4^+^ T cell subsets to pulmonary function in patients with IPF. Our novel findings imply that this particular recruitment and expansion of CCR4^+^ CD4^+^ T cells in IPF lungs modulates the pro-fibrotic effects of CCR6^+^ CD4^+^ T cells within the diseased lungs, and may be an active mechanism to preserve lung function. Our results are consistent with a recently terminated IPF clinical trial, which demonstrated that monoclonal antibody inhibition of CCL2, a CCR4 chemokine ligand, showed no benefit and may even be detrimental (55). Together, our findings demonstrate that augmentation of chemokines that recruit protective CCR4^+^ CD4^+^ T cells, while blocking chemokines that recruit detrimental CCR6^+^ CD4^+^ T cells, may constitute a novel approach for IPF therapy.

## Author Contributions

Conception and design: AA, CH, CB, and AS. Data acquisition, analysis, and interpretation: AA, CH, CB, MJ, MS, KB, MC, MS, IN, and AS. Drafting the manuscript for important intellectual content: AA, CH, CB, MJ, KB, MS, MC, MS, IN, and AS. Additionally, all the authors had substantial contributions to critically revising this manuscript for important intellectual content, have provided final approval of the version to be published, and agree to be accountable for all the aspects of this work in ensuring that questions related to the accuracy or integrity of any part of this work are appropriately investigated and resolved.

## Conflict of Interest Statement

The authors declare that the research was conducted in the absence of any commercial or financial relationships that could be construed as a potential conflict of interest.
