# Supplementary material for: Skewed Lung CCR4 to CCR6 CD4+ T Cell Ratio in Idiopathic Pulmonary Fibrosis Is Associated with Pulmonary Function
Source: Front Immunol. 2016 Nov 23;7:516. doi: 10.3389/fimmu.2016.00516 (PMC5120085; doi:10.3389/fimmu.2016.00516)
Supplement: Supplementary file 1 [file Data_Sheet_1.PDF]

Skewed Lung CCR4 to CCR6 CD4 T cell ratio in Idiopathic Pulmonary Fibrosis is Associated with Better Lung Function

Ayodeji Adegunsoye[1]*, Cara L. Hrusch[1,2]*, Catherine A. Bonham[1]*, Mohammad R. Jaffery [1], Kelly M. Blaine[1], Meghan Sullivan[1], Matthew M. Churpek[1], Mary E. Strek[1], Imre Noth[1], Anne I. Sperling[1,2]

ONLINE DATA SUPPLEMENT

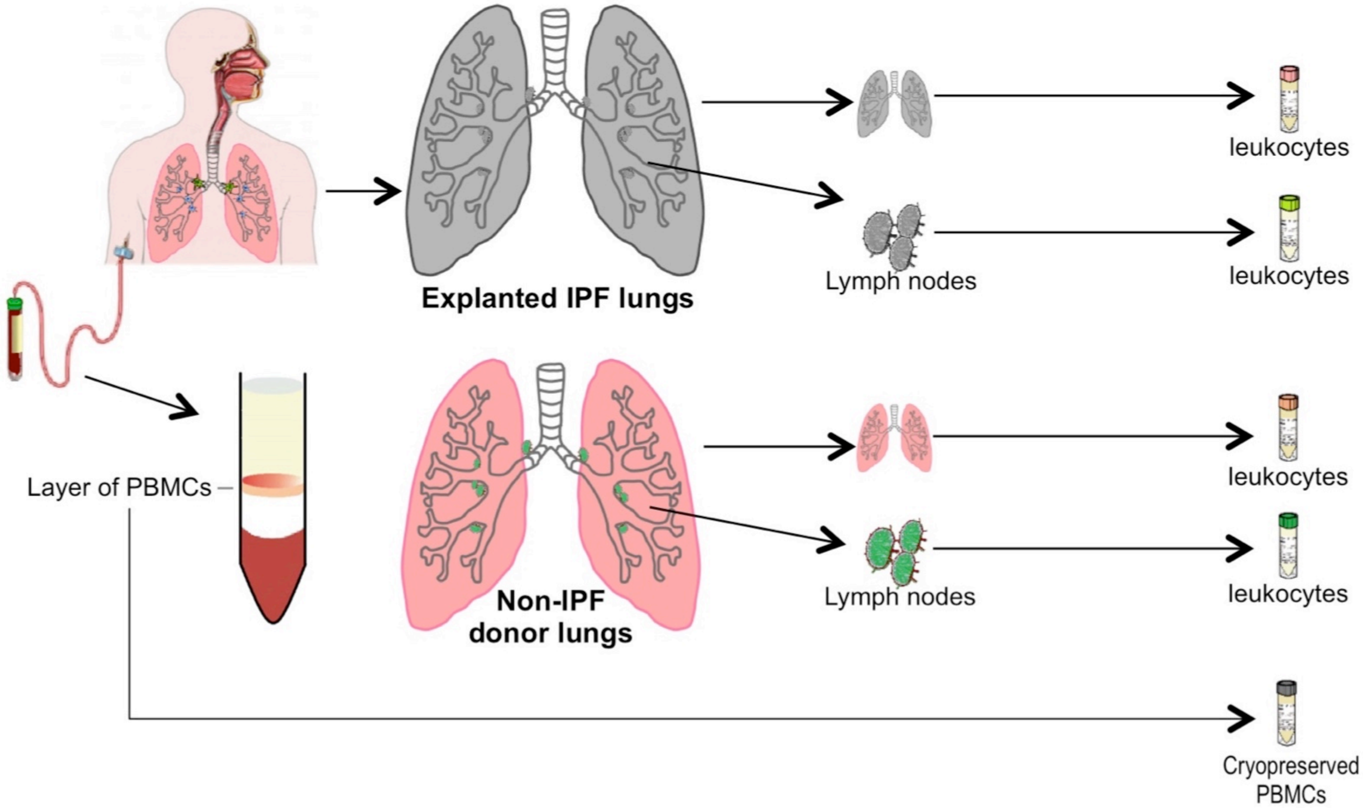

e-Supplement S1. Sample and data acquisition. Lung tissue, lung lymph node tissue and blood were collected from patients with IPF (n=9; blood samples not available in one patient with IPF) and from control subjects with no fibrotic lung disease (n=13). All samples were processed for extraction of mononuclear leukocytes.

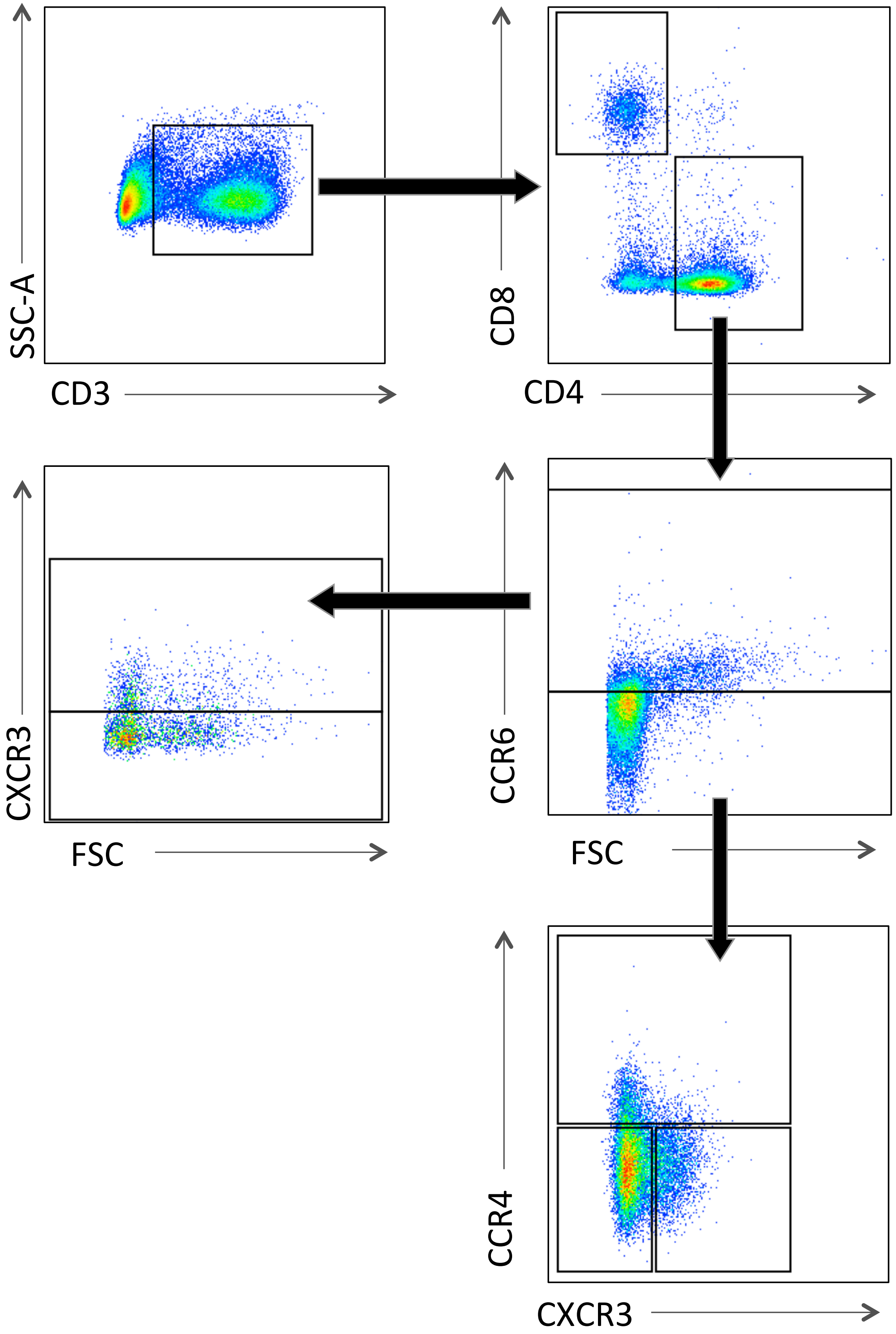

e-Supplement S2. Gating strategy for T-cell polarization in IPF. CD3^{+} CD4^{+} T cells were first separated into CCR6^{-} or CCR6^{+} cells. These subsets were then divided into CXCR3^{+}CCR4^{-}CCR6^{-} or CCR4^{+}CCR6^{-} cells based on CCR4 and CXCR3 expression; and CCR6^{+}CXCR3^{-} or CCR6^{+}CXCR3^{+} cells.

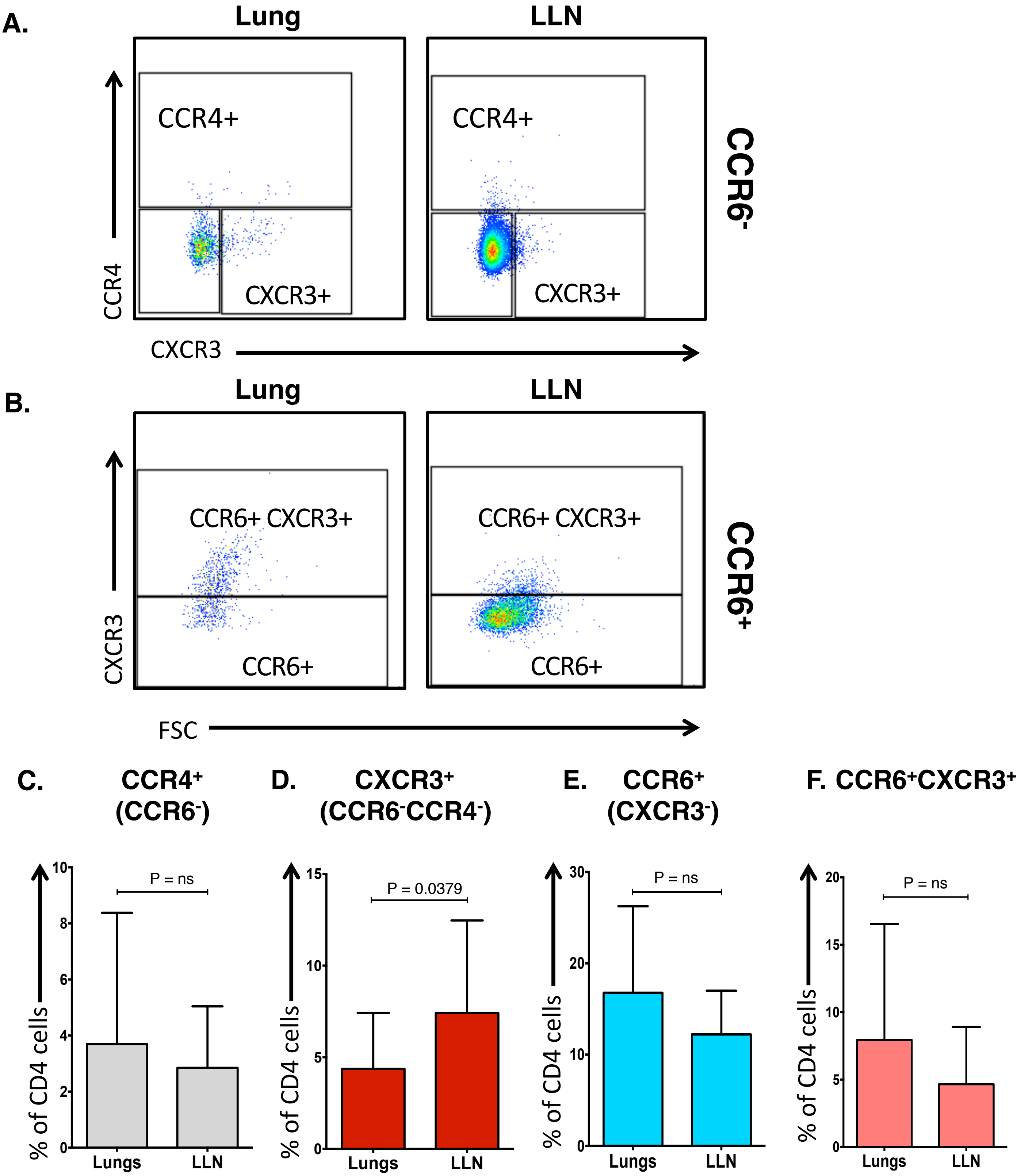

e-Supplement S3. Phenotypic distribution of CD4+ CCR6- T cells and CD4+ CCR6+ T cells across tissue locations in controls. CCR6- lung lymph nodes (LLN) cells have a predominant expression of CXCR3 (A, D). The expression of CCR4 in CCR6- lung cells did not differ when compared to LLN cells (A, C). No differences were observed in CXCR3 expression when comparing CCR6+ lung cells to CCR6+ LLN cells (B, E, F). Blood cells were not available in control patients for comparison with lung or LLN cells. Significance determined by the paired t-test.

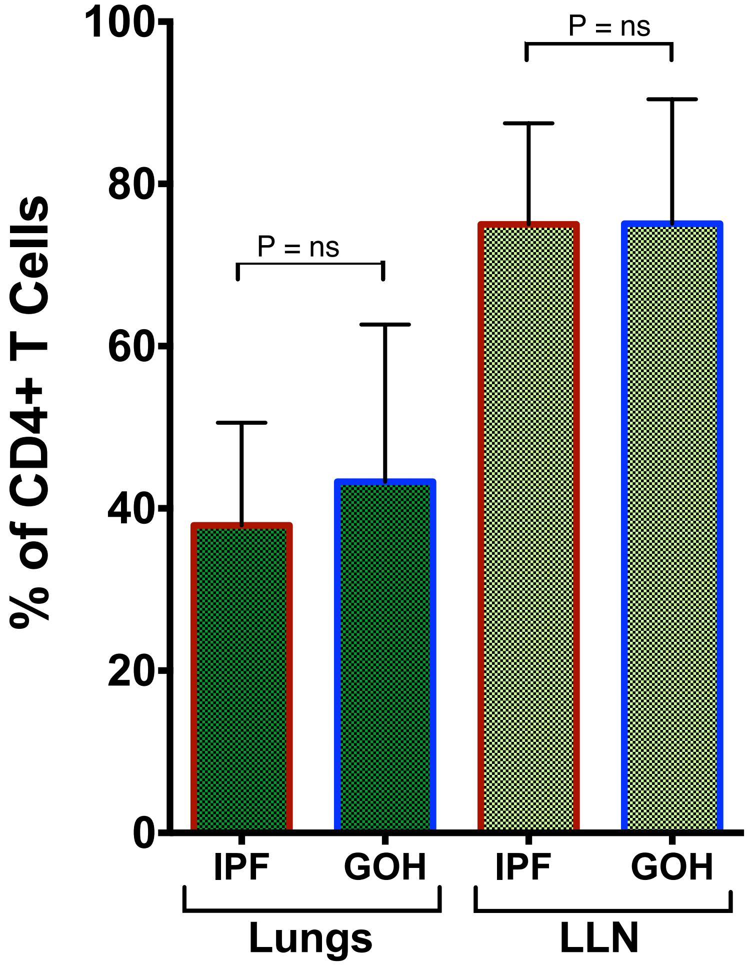

e-Supplement S4. Phenotypic distribution of CD4⁺ CCR7⁺ T cells across tissue locations. CCR7⁺ distribution in lungs and lung lymph nodes (LLN) cells do not differ between IPF and control subjects. Significance determined by the Mann-Whitney test.

E-supplement Table 1. Correlation of FVC and DLCO with lung and lung lymph nodes CD4 T cell subsets at time of lung explant in IPF patients

CD4 T cell subset (% of CD4)	FVC Correlation (R-value)	p-value	DLCO Correlation (R-value)	p-value
Lungs				
$CCR4^{+}(CCR6^{-})$	0.65	0.06	0.55	0.12
$CXCR3^{+}(CCR6^{-}CCR4^{-})$	0.43	0.24	0.06	0.88
$CCR6^{+}(CXCR3^{-})$	0.07	0.85	0.58	0.10
$CCR6^{+}(CXCR3^{+})$	-0.39	0.24	0.12	0.75
Lung Lymph Nodes				
$CCR4^{+}(CCR6^{-})$	0.66	0.05	0.47	0.20
$CXCR3^{+}(CCR6^{-}CCR4^{-})$	-0.16	0.67	0.34	0.37
$CCR6^{+}(CXCR3^{-})$	0.34	0.37	0.41	0.27
$CCR6^{+}(CXCR3^{+})$	-0.13	0.73	0.47	0.20
Blood				
$CCR4^{+}(CCR6^{-})$	0.36	0.38	0.15	0.73
$CXCR3^{+}(CCR6^{-}CCR4^{-})$	-0.31	0.46	0.34	0.40
$CCR6^{+}(CXCR3^{-})$	-0.00	0.99	0.69	0.06
$CCR6^{+}(CXCR3^{+})$	-0.33	0.43	0.44	0.27
Ratio				
Lungs $CCR4^{+}$: $CCR6^{+}$	0.86	0.003	0.17	0.66
Lung Lymph node $CCR4^{+}$: $CCR6^{+}$	0.61	0.08	0.46	0.21
Blood $CCR4^{+}$: $CCR6^{+}$	0.23	0.58	-0.56	0.15

R-values determined by utilizing the Pearson correlation test.
